# Notes and News

**Published:** 1903-01-03

**Authors:** 


### Notes and News.

Mr. Jonathan Hutchinson has gone to India and Ceylon to investigate the distribution of leprosy and to further test his opinion that badly cured fish is largely responsible for the spread of the disease.

The League of Mercy has handed over to King Edward's Hospital Fund a cheque for £8,000, as the result of its collection of small subscriptions of one shilling and upwards in 1902.

A County Ball will be held in aid of the Sussex County Hospital on Wednesday, January 14th, at Brighton. The first list of patrons includes the Duke of Cambridge. The hospital is in debt to the amount of £10,000.

A prize for fifth-year students at St. Thomas's Hospital has been established in memory of Dr. Seymour Toller, formerly assistant physician, by his parents. On their part the Governors of St. Thomas's will name the new children's ward the Seymour Toller Ward.

Three courses of advanced lectures will be delivered in the Physiological Laboratory of the University of London, commencing respectively on January 20th, 22nd, and 23rd, and continuing week by week. On successive Tuesdays the lectures will be "On the Chemistry of Muscle and Nerve," by W. D. Halliburton, M.D., F.R.S.; on Wednesdays "On the Circulation," by T. G. Brodie, M.D.; and on Thursdays "On the Action of Anæsthetics and Narcotics," by A. D. Waller, M.D., F.R.S. The lectures are addressed to advanced students, and are arranged to meet the requirements of candidates for honours in physiology at the University. Cards of admission may be obtained on application to the Academic Registrar.

The East Ham Council have opened a new Isolation Hospital, constructed of iron, at a cost of about £2,800, to accommodate 20 diphtheria and 20 typhoid cases.

The Chelsea Hospital for Women has received a bequest of £1,000 from the estate of the late Mr. Francis Thomas Freeman allotted under the discretionary powers of the executors.

The new Infirmary and Nurses' Home at the Richmond Workhouse has been opened. The infirmary is for the accommodation of 174 patients. In each of the two main blocks wards for 24 beds in each are distributed over three floors. The cost has been £40,000.

With the commencement of this year a new weekly paper entitled *Education*, dealing with the work of the authorities under the Education Bill, is being issued by the St. Bride's Press. Let us hope that it will take reasonable views as to the many medical questions which are involved in compulsory education.

The news that an architect has been selected to plan the King's Sanatorium for Consumption will be received with interest. Mr. H. Percy Adams has had much experience in hospital planning, and done some admirable work. He will have the advantage of the medical opinion elicited by the Prize Competition which was arranged for members of the medical profession.

The Coronation Gift Fund in connection with the British Home and Hospital for Incurables to the Queen, will be closed on December 31st next, and all intending subscribers are asked to kindly forward their donations at once to the secretary. The signatures of all the subscribers to the Fund will be placed in an album and offered for her Majesty's gracious acceptance, cheques and postal orders to be crossed "Barclay and Co." and sent to Edgar Penman, Secretary; Offices, 72 Cheapside, E.C. There are 79 inmates in the Home, and 348 pensioners received £20 per annum.

Mr. W. W. Astor has given £50,000 to the Hospital for Sick Children, Great Ormond Street, as a fund for the building of a new out-patient department, to be dedicated to the memory of his little daughter Gwendoline Astor, who died this autumn. The very inefficient condition through want of accommodation of the present out-patients' department in the basement of the hospital has caused much anxiety to the committee, and frequent complaints by the visitors from King Edward's Fund. Mr. Astor's most generous gift will enable the committee to put the department into a thoroughly efficient condition, and, as several suitable sites have been offered to them, it is only a matter of a few months before this most desirable improvement will be effected. We are glad to note that the authorities of this hospital acknowledge the "valuable assistance which criticism from King Edward's Fund brings forth." We notice also that where the administration is conducted with intelligence and with singleness of purpose this recognition is forthcoming.

